# Associations of hedonic and eudaimonic orientations with subjective experience and objective functioning in academic settings: The mediating roles of academic behavioral engagement and procrastination

**DOI:** 10.3389/fpsyg.2022.948768

**Published:** 2022-08-11

**Authors:** Hezhi Chen, Zhijia Zeng

**Affiliations:** ^1^Department of Psychology, School of Education, Zhejiang International Studies University, Hangzhou, China; ^2^Mental Health Education Center, Zhejiang University of Finance and Economics, Hangzhou, China

**Keywords:** happiness orientation, affective well-being, academic achievement, academic engagement, procrastination

## Abstract

The question of how the pursuit of happiness affects an individual’s actual well-being has received much scholarly attention in recent years. However, few studies have investigated the associations of happiness orientation with people’s subjective experience and objective functioning simultaneously. The current research examines the possibility that hedonic and eudaimonic orientations have different relationships with college students’ affective well-being and academic achievement, while taking into consideration the behavioral mechanism that underlies the process. We conducted online surveys to collect data including hedonic and eudaimonic orientations at the beginning of the semester and academic behavioral engagement, procrastination, and affective well-being at the end of the semester with a final sample of 566 Chinese undergraduate students. Their official grade point average for the semester was extracted from the university records system. The results confirmed that overall hedonic orientation was negatively associated with affective well-being and academic achievement, whereas eudaimonic orientation was positively associated with these outcomes. The study further found that both academic behavioral engagement and procrastination played mediating roles in the associations of happiness orientation with positive affect and academic achievement. However, only procrastination mediated the relationship between happiness orientation and negative affect. Theoretical and practical implications were discussed.

## Introduction

Happiness is one of the common goals for human beings. Nevertheless, people have different ideas about what constitutes a good life and how to enhance their well-being. There are two fundamental orientations to happiness: hedonic orientation (seeking enjoyment and pleasure) and eudaimonic orientation (seeking personal growth and a meaningful life) (Huta and Ryan, 2010). Early research proposes that a “full life” orientation, which refers to both hedonia and eudaimonia, ensures the pinnacle of happiness (Peterson et al., 2005; Huta and Ryan, 2010). In contrast, recent studies suggest that the pursuit of happiness, especially hedonia, may not improve individual well-being and sometimes could bring about the opposite effect (Sheldon et al., 2019). Despite the contradictory findings, most previous research focused on the association of happiness orientation with subjective experience but neglected individuals’ objective functioning. In addition, the mechanism behind the influence of happiness orientation is also not fully explored. To address these research gaps, the present study aimed to examine the associations of pursuing hedonia and eudaimonia with college students’ subjective affective experience and objective academic achievement, as well as the behavioral mechanism underlying the process.

### Happiness orientation, affective well-being, and academic achievement

The “full life” hypothesis, first proposed by Peterson et al. (2005) and later developed by Huta and Ryan (2010), posits that hedonic and eudaimonic orientations contribute to different aspects of well-being. Thus, a combination of them leads to the highest level of well-being. Several studies have provided evidence supporting this theory (Peterson et al., 2005; Huta and Ryan, 2010; Henderson et al., 2013). For instance, in one of the earliest studies on the relationship between happiness orientation and individual well-being, pleasure, engagement, and meaning orientations were all positively related to life satisfaction. Furthermore, individuals that experience high levels of all three aforementioned orientations simultaneously reported the greatest life satisfaction (Peterson et al., 2005). Similarly, Huta and Ryan (2010) found that hedonic orientation has stronger associations with hedonic aspects of well-being; eudaimonic orientation has stronger associations with eudaimonic aspects of well-being; both orientations were equally related to general life satisfaction. These findings have also been replicated with samples from various countries such as China and Japan (Chen, 2010; Asano et al., 2021).

Despite the above findings, recent empirical research has suggested that the well-being benefits of pursuing happiness are not guaranteed (Joshanloo and Jarden, 2016; Jia et al., 2021). In particular, hedonia may be associated with decreased individual well-being (Yang et al., 2017a; Sheldon et al., 2019; Lin and Chan, 2020; Zeng and Chen, 2020). The distinct associations of hedonic and eudaimonic orientations with well-being outcomes may be accounted for by two significant differences between them (Huta, 2015). First, hedonia is related to people’s short-term needs, whereas eudaimonia is associated with long-term objectives. Second, hedonic motives prompt people to chase what makes them feel good, while eudaimonic intent guides people to do what is right or needs to be done. In particular, excessive or unbalanced hedonic motives are likely to make people focus too much on momentary pleasure, hindering them from fulfilling long-term goals, and ultimately impairing their well-being. A few recent studies have documented the negative associations between hedonic orientation and the subjective experience of well-being (Sheldon et al., 2019; Lin and Chan, 2020; Zeng and Chen, 2020). In contrast, eudaimonia is regarded as a high path to happiness as it generally brings more positive outcomes (Schueller and Seligman, 2010).

While individual well-being includes both how people feel or evaluate their life and how they actually do or perform in their life, previous research has focused on the association of happiness orientation with people’s subjective experience and much neglected its relationship with people’s objective functioning such as achievement in an academic setting. To our knowledge, only one study has investigated this issue and found that college students’ hedonic orientations negatively related to their academic performance, although the effect was only marginally significant due to the limited sample size (Kryza-Lacombe et al., 2019).

### Academic behavioral engagement and procrastination as mediators

Happiness orientation may affect students’ affective well-being and academic achievement through certain behaviors. In this study, we will examine academic behavioral engagement and procrastination as the two mediators. Academic behavioral engagement refers to the extent and manner in which students are involved in academic activities (Fredricks et al., 2004). Among these, a persistent effort has been recognized as one of the core elements (Miller et al., 1996). Effort refers to the amount of time or energy expended in academic tasks, and persistence refers to continuous efforts toward solving problems rather than giving up, especially when confronted with challenges (Elliot et al., 1999). In contrast, academic procrastination refers to delay in starting or completing academic tasks, despite the awareness of potential negative outcomes (Klassen et al., 2008). Approximately over half of college students procrastinate, which suggests that it may be the most common approach used by them to disengage from academic work (Steel, 2007; Özer et al., 2009).

While few studies have investigated the associations of happiness orientation with academic behavioral engagement and procrastination, substantial evidence indicates that hedonic, and eudaimonic orientations are likely to play different roles in predicting these two types of behavior. Focus on the future positively relates to students’ dedication to academic work (Denovan et al., 2020), and negatively relates to procrastination (Chen and Chang, 2016). In contrast, prioritizing momentary pleasure over future consequences leads to more procrastination (Ferrari and Díaz-Morales, 2007), and prevents students from exerting persistent efforts on studying (Horstmanshof and Zimitat, 2007; Barnett et al., 2020). Moreover, individuals with strong hedonic orientations were found to have lower levels of self-control (Anic and Tonèiæ, 2013; Gentzler et al., 2021). Self-control is of great importance in enabling students to engage in academic activities, especially when they are challenging and unpleasant (King and Gaerlan, 2014). Lacking self-control is one of the most crucial reasons for procrastination (Steel, 2007; Klassen et al., 2008). Therefore, it is reasonable to assume that students with a strong hedonic orientation will show less academic behavioral engagement and more procrastination, whereas students with a strong eudaimonic orientation will display the reverse.

Further, ample evidence has shown that academic behavioral engagement is beneficial to college students’ affective well-being and academic achievement. Unsurprisingly, students who engaged more in academic activities achieved better performance (Chase et al., 2014; van Rooij et al., 2017). Active participation in study and better achievement also satisfied students’ basic psychological needs (Wang et al., 2019), thus, eliciting positive emotions and decreasing negative emotions (Datu and King, 2018; Li et al., 2020). In contrast, extensive research has demonstrated that procrastination is generally maladaptive, with a wide range of consequential outcomes. A meta-analysis has shown that overall procrastination is negatively associated with students’ academic performance (Kim and Seo, 2015). Moreover, studies have consistently found that procrastinators tend to experience more stress (Sirois, 2014), more negative emotions such as anxiety and guilt (Sirois et al., 2019), and fewer positive emotions (Sirois and Giguère, 2018).

### The present study

The present study aims to examine (a) the associations of hedonic and eudaimonic orientations with college students’ affective well-being and academic achievement, and (b) whether academic behavioral engagement and procrastination play mediating roles in the process. Based on the literature review, we propose the following hypotheses.

*Hypothesis 1*: Hedonic orientation is negatively associated with affective well-being and academic achievement.

*Hypothesis 2*: Eudaimonic orientation is positively associated with affective well-being and academic achievement.

*Hypothesis 3*: Academic behavioral engagement and procrastination mediate the associations of hedonic and eudaimonic orientations with affective well-being and academic achievement.

## Materials and methods

### Participants and procedure

In the second week of the first semester, all first-year undergraduate students from a public university in northwest China attended a general psychological online survey for course credits. During the survey, the students completed the scale assessing hedonic and eudaimonic orientations, and other scales unrelated to the current study. Approximately 3 months later, a subsample of 606 students was recruited to attend another online survey during class. Data regarding academic behavioral engagement, procrastination, and affective well-being were collected. Online informed consent was obtained from all participants. In the second survey, participants were compensated by conducting a draw with a two percent chance of winning a ¥50 (approximately $8) gift card. Thirty participants who did not attend the first wave survey along with 10 other participants who failed on attention-check items were removed from the formal analysis, leaving the final sample of 566 students (374 women, 192 men, *M*_age_ = 18.45, *SD* = 0.64). At the end of the semester, the official grade point average (GPA) of the first semester was extracted from the university records system as an indicator of academic achievement. Student identity numbers were used to pair the data collected in the three waves.

### Measures

All the scales used in the current study were translated from English to Chinese and back to English by two bilingual colleagues, respectively. When there were discrepancies between the original English version and the back-translated English version, those items were further modified after discussion by the two authors.

### Hedonic and eudaimonic orientations

The two kinds of happiness orientations were measured using the Hedonic and Eudaimonic Motives for Activities-Revised Scale (Huta, 2016). This instrument utilizes five items to assess hedonic orientation (e.g., “seeking pleasure”) and five items to assess eudaimonic orientation (e.g., “seeking to use the best in yourself”). Participants indicated the degree to which they used each motive in their daily activities on a seven-point Likert scale ranging from 1 (not at all) to 7 (very much). The scale has been found to have good psychometric properties in Chinese samples (Li et al., 2021). In the present study, Cronbach’s α coefficient was 0.80 for hedonic orientation and 0.81 for eudaimonic orientation.

### Academic behavioral engagement

Academic behavioral engagement was measured using four items from the effort subscale of the Motivated Strategies for Learning Questionnaire (Pintrich et al., 1993) (e.g., “I work hard to do well in this class even if I don’t like what we are doing”) and another four items from the Mastery Behaviors Questionnaire (Porter et al., 2020) (e.g., “When I am doing a challenging problem in school, I do not give up until I have found a solution”). Participants indicated their agreement with each item on a seven-point Likert scale ranging from 1 (strongly disagree) to 7 (strongly agree) based on their experience in the semester. Confirmatory factor analysis (CFA) showed that one-factor model had an appropriate fit index [χ2 (18) = 82.23, χ2/df = 4.57, CFI = 0.96, TLI = 0.94, RMSEA = 0.079, SRMR = 0.033]. Cronbach’s α coefficient of the scale was 0.84.

### Procrastination

Procrastination was measured using the Tuckman Procrastination Scale (Tuckman, 1991), which was designed for college students. The scale comprises 16 items (e.g., “I needlessly delay finishing jobs, even when they are important”). Each item was rated on a seven-point Likert scale ranging from 1 (strongly disagree) to 7 (strongly agree), according to the participants’ experience in the semester. Cronbach’s α coefficient of the scale was 0.91.

### Affective well-being

Affective well-being was measured using the Scale of Positive and Negative Experience (Diener et al., 2010). The scale comprises six items related to positive experience (e.g., “pleasant”) and six items related to negative experience (e.g., “unpleasant”). Participants reported the frequency of each feeling in the past four weeks on a five-point Likert scale from 1 (very rarely or never) to 5 (very often or always). This scale has been validated with Chinese samples in previous studies (Li et al., 2013). In the present study, Cronbach’s α values were 0.92 and 0.89 for positive affect and negative affect, respectively.

### Academic achievement

Academic achievement was operationalized as the official GPA in the first semester. The GPA scores ranged from 0.00 to 5.00, with higher scores indicating better academic achievement.

### Statistical analysis

Data were analyzed using SPSS 24. First, we calculated the descriptive statistics and correlations of the study variables. Next, we conducted multiple regression analyses for each component of the parallel mediation of academic behavioral engagement and procrastination in the associations of hedonic and eudaimonic orientations with academic achievement, positive affect, and negative affect. Moreover, we used SPSS macro PROCESS (model 4) and performed mediation analyses with 5,000 bootstrap samples to estimate the 95% confidence interval (CI) (Preacher and Hayes, 2004; Hayes, 2017). The indirect effect was considered significant if the 95% CI did not contain zero. We also controlled gender and age in the analysis. The main results did not change whether they were included as control variables or not.

## Results

### Descriptive statistics and correlations

Descriptive statistics and correlations between variables are presented in Table 1. Hedonic orientation was negatively related to positive affect (*r* = −0.09, *p* = 0.030) but not related to GPA (*r* = −0.06, *p* = 0.146) or negative affect (*r* = 0.08, *p* = 0.051). Eudaimonic orientation was positively related to GPA (*r* = 0.13, *p* = 0.003) and positive affect (*r* = 0.14, *p* = 0.001) but not related to negative affect (*r* = −0.07, *p* = 0.109). The results also found that hedonic orientation was negatively related to academic behavioral engagement (*r* = −0.09, *p* = 0.041) and positively related to procrastination (*r* = 0.12, *p* = 0.004). In contrast, eudaimonic orientation was positively related to academic behavioral engagement (*r* = 0.32, *p* < 0.001) and negatively related to procrastination (*r* = −0.26, *p* < 0.001). Furthermore, academic behavioral engagement was positively correlated with GPA (*r* = 0.29, *p* < 0.001) and positive affect (*r* = 0.27, *p* < 0.001), and negatively correlated with negative affect (*r* = −0.16, *p* < 0.001). Procrastination was negatively correlated with GPA (*r* = −0.24, *p* < 0.001) and positive affect (*r* = −0.26, *p* < 0.001) and positively correlated with negative affect (*r* = 0.32, *p* < 0.001).

**TABLE 1:** Means, standard deviations, and zero-order correlations of the study variables.

	1	2	3	4	5	6	7
1. Hedonic orientation	−						
2. Eudaimonic orientation	0.24***	−					
3. Academic behavioral engagement	−0.09*	0.32***	−				
4. Procrastination	0.12**	−0.26***	−0.56***	−			
5. Grade average point	−0.06	0.13**	0.29***	−0.24***	−		
6. Positive affect	−0.09*	0.14**	0.27***	−0.26***	0.08	−	
7. Negative affect	0.08	−0.07	−0.16***	0.32***	0.02	−0.38***	−
Mean	4.41	4.96	4.98	3.59	3.47	3.58	2.69
Standard deviation	1.00	1.02	0.89	0.96	0.56	0.63	0.67

*N* = 566; *p < 0.05, **p < 0.01, ***p < 0.001.

### Mediating analyses

**FIGURE 1:**
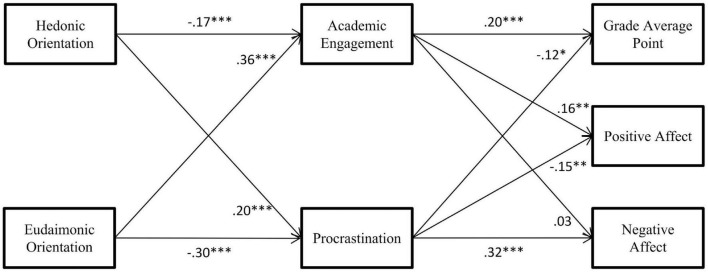
Indirect effects of happiness orientation on well-being outcomes through academic behavioral engagement and procrastination. The numbers represent standardized estimated coefficients. Participants’ gender and age were allowed to predict academic behavioral engagement, procrastination, grade average point, positive affect, and negative affect while omitted in the figure. The direct effects of happiness orientation (which were all non-significant) were also omitted in the figure. *p < 0.05, **p < 0.01, ***p < 0.001.

A series of hierarchical multiple regression analyses were conducted to investigate the indirect effects of happiness orientation on well-being outcomes (a summary was presented in Figure 1). First, we tested the mediation effects of hedonic and eudaimonic orientations on GPA *via* academic behavioral engagement and procrastination. The results of the regression analyses are presented in Table 2. Overall, hedonic orientation was negatively related to GPA (ß = −0.09, *p* = 0.038), while eudaimonic orientation was positively related to GPA (ß = 0.16, *p* < 0.001). Moreover, both hedonic and eudaimonic orientations had significant effects on the two mediators. Specifically, hedonic orientation was negatively related to academic behavioral engagement (ß = −0.17, *p* < 0.001) and positively related to procrastination (ß = 0.20, *p* < 0.001). Meanwhile, eudaimonic orientation was positively related to academic behavioral engagement (ß = 0.36, *p* < 0.001) and negatively related to procrastination (ß = −0.30, *p* < 0.001). When hedonic and eudaimonic orientations were controlled, higher academic behavioral engagement was related to better GPA (ß = 0.20, *p* < 0.001) and higher procrastination was related to worse GPA (ß = −0.12, *p* = 0.017). The bootstrapping procedures confirmed the mediating roles of academic behavioral engagement and procrastination in the relationship between the two types of orientation and GPA (see Table 3). Hedonic orientation had negative indirect effects on GPA through academic behavioral engagement [effect = −0.033, 95%CI (−0.061, −0.012)] and procrastination [effect = −0.023, 95%CI (−0.045, −0.004)]. Eudaimonic orientation had positive indirect effects on GPA through academic behavioral engagement [effect = 0.071, 95%CI (0.035, 0.110)] and procrastination [effect = 0.035; 95%CI (0.009, 0.064)].

**TABLE 2:** Results of regression analyses.

	*B*	*ß*	*p*	*R* ^2^
**Dependent variable: Academic behavioral engagement**				0.13
Gender	−0.08	−0.04	*ns*	
Age	−0.01	−0.01	*ns*	
Hedonic orientation	−0.15	−0.17	< 0.001	
Eudaimonic orientation	0.32	0.36	< 0.001	
**Dependent variable: Procrastination**				0.10
Gender	−0.06	−0.03	*ns*	
Age	−0.07	−0.05	*ns*	
Hedonic orientation	0.19	0.20	< 0.001	
Eudaimonic orientation	−0.28	−0.30	< 0.001	
**Dependent variable: Grade average point**				
Model 1				0.06
Gender	−0.22	−0.19	< 0.001	
Age	−0.04	−0.05	*ns*	
Hedonic orientation	−0.05	−0.09	0.038	
Eudaimonic orientation	0.09	0.16	< 0.001	
Model 2				0.13
Gender	−0.21	−0.18	< 0.001	
Age	−0.05	−0.05	*ns*	
Hedonic orientation	−0.02	−0.03	*ns*	
Eudaimonic orientation	0.03	0.06	*ns*	
Academic behavioral engagement	0.12	0.20	< 0.001	
Procrastination	−0.07	−0.12	0.017	
**Dependent variable: Positive affect**				
Model 1				0.04
Gender	−0.04	−0.03	*ns*	
Age	0.02	0.02	*ns*	
Hedonic orientation	−0.08	−0.13	0.002	
Eudaimonic orientation	0.10	0.17	< 0.001	
Model 2				0.10
Gender	−0.04	−0.03	*ns*	
Age	0.02	0.02	*ns*	
Hedonic orientation	−0.05	−0.08	*ns*	
Eudaimonic orientation	0.04	0.07	*ns*	
Academic behavioral engagement	0.11	0.16	0.001	
Procrastination	−0.10	−0.15	0.003	
**Dependent variable: Negative affect**				
Model 1				0.03
Gender	−0.14	−0.10	0.018	
Age	−0.04	−0.03	*ns*	
Hedonic orientation	0.07	0.11	0.012	
Eudaimonic orientation	−0.06	−0.08	*ns*	
Model 2				0.11
Gender	−0.13	−0.09	0.027	
Age	−0.02	−0.02	*ns*	
Hedonic orientation	0.03	0.05	*ns*	
Eudaimonic orientation	0.00	0.00	*ns*	
Academic behavioral engagement	0.02	0.03	*ns*	
Procrastination	0.23	0.32	< 0.001	

**TABLE 3:** Indirect effects of happiness orientation on GPA, positive affect, and negative affect.

	Hedonic orientation		Eudaimonic orientation	
		
	Effect	95% CI	Effect	95% CI
**Dependent variable: GPA**				
Academic behavioral	−0.033	−0.061, −0.012	0.071	0.035, 0.110
engagement				
Procrastination	−0.023	−0.045, −0.004	0.035	0.009, 0.064
Total indirect effects	−0.056	−0.088, −0.028	0.106	0.073, 0.142
**Dependent variable: Positive affect**				
Academic behavioral	−0.027	−0.053, −0.008	0.058	0.020, 0.099
engagement				
Procrastination	−0.028	−0.056, −0.006	0.043	0.011, 0.078
Total indirect effects	−0.056	−0.088, −0.028	0.102	0.065, 0.141
**Dependent variable: Negative affect**				
Academic behavioral	−0.005	−0.026, 0.017	0.010	−0.032, 0.052
engagement				
Procrastination	0.063	0.030, 0.102	−0.097	−0.141, −0.059
Total indirect effects	0.059	0.025, 0.097	−0.088	−0.133, −0.045

The results showed a similar pattern when positive affect was used as the dependent variable. Overall, hedonic orientation was negatively related to positive affect (ß = −0.13, *p* = 0.002), while eudaimonic orientation was positively related to positive affect (ß = 0.17, *p* < 0.001). In addition, higher academic behavioral engagement was related to more positive affect (ß = 0.16, *p* = 0.001) and higher procrastination was related to less positive affect (ß = −0.15, *p* = 0.003) when hedonic and eudaimonic orientations were controlled. The bootstrapping analysis showed that hedonic orientation had negative indirect effects on positive affect through academic behavioral engagement [effect = −0.027, 95%CI (−0.053, −0.008)] and procrastination [effect = −0.028, 95%CI (−0.056, −0.006)]. In contrast, eudaimonic orientation had positive indirect effects on positive affect through academic behavioral engagement [effect = 0.058, 95%CI (0.020, 0.099)] and procrastination [effect = 0.043, 95%CI (0.011, 0.078)].

When using happiness orientation to predict negative affect, only hedonic orientation showed a positive association (ß = 0.11, *p* = 0.012). More importantly, only one mediator, procrastination, was positively related to negative affect (ß = 0.32, *p* < 0.001) after controlling for hedonic and eudaimonic orientations. The bootstrapping procedures further indicated that hedonic orientation had a positive indirect effect on negative affect only through procrastination [effect = 0.063, 95%CI (0.030, 0.102)], and eudaimonic orientation had a negative indirect effect on negative affect through the same mediator [effect = −0.097, 95%CI (−0.141, −0.059)].

## Discussion

This study examined the associations of hedonic and eudaimonic orientations on college students’ subjective experience and objective functioning in academic settings and explored the behavioral mechanism underlying the process. Overall, students’ hedonic orientation was negatively associated with affective well-being and academic achievement, whereas eudaimonic orientations were positively associated with these outcomes. Furthermore, the results confirmed the hypothesized mediation roles of academic behavioral engagement and procrastination in the associations of happiness orientation with affective well-being and academic achievement.

### Association of happiness orientation with well-being outcomes

This study first examined the relationship between happiness orientation and two different aspects of well-being outcomes. As expected, the results revealed that hedonic, and eudaimonic orientations had distinct associations with college students’ affective well-being and academic achievement. Students with a strong eudaimonic orientation experienced more positive affect and less negative affect and obtained better academic achievement; in contrast, hedonic orientation was related to worse affective well-being and academic performance.

Previous research has shown inconsistent results regarding the association of happiness orientation with individual well-being. The full life hypothesis (Peterson et al., 2005; Huta and Ryan, 2010), which posits that both pursuing hedonia and eudaimonia are related to an increase in actual well-being, was not fully supported in the current study. Instead, in accordance with some recent studies (Sheldon et al., 2019; Lin and Chan, 2020; Chen and Zeng, 2021), our findings suggested that the pursuit of hedonia could be related to a decrease in individuals’ well-being. More importantly, this study expands previous research by extending the negative outcomes of hedonia from subjective experience to objective functioning. Previous research has focused on the relationship between happiness orientation and well-being outcomes such as life satisfaction. However, happiness should not be limited to subjective experience but also include how well an individual is actually doing in real life. Education is one of the most crucial domains for college students and is the foundation for future success. This study provides evidence that the pursuit of hedonia is associated with poor performance in academic settings, while the pursuit of eudaimonia generally relates to improved academic achievement.

### Mediating roles of academic behavioral engagement and procrastination

The study examined the behavioral mechanism underlying the associations of happiness orientation with affective well-being and academic achievement. The results revealed that both academic behavioral engagement and procrastination mediated the associations of happiness orientation with positive affect and academic achievement. However, only procrastination mediated the relationship between happiness orientation and negative affect. In other words, actively engaging in academic activities enhanced positive affect but did not help to diminish negative experiences. Instead, negative affect was primarily influenced by the extent to which individuals use maladaptive behaviors such as procrastination. This is consistent with theoretical traditions of positive psychology which claim the factors affecting positive emotions are different from those affecting negative emotions.

This study provides a deeper understanding of how hedonic and eudaimonic orientations exert different effects on individual well-being. According to the motive-behavior-experience model (Huta, 2015), hedonic and eudaimonic orientations motivate people to engage in more hedonic and eudaimonic behaviors respectively, and this affects an individual’s well-being. While theoretically researchers have suggested that excessive hedonic orientation may also lead to maladaptive hedonic behaviors, empirically only a few studies have investigated these potential links (Yang et al., 2017a; Giuntoli et al., 2021). The current findings indicate that happiness orientation is closely related to people’s behavior in academic settings. Education is important but generally not enjoyable, and sometimes can be challenging, tough, and even exhausting. When confronted with difficulties in studying, students with strong hedonic orientations are likely to exert less effort or choose to postpone starting work for momentary relaxation. In contrast, the pursuit of eudaimonia encourages students to persistently perform their tasks and prevents them from procrastinating. These different behavioral patterns, which are largely related to happiness orientation, further determine how well students actually perform and the extent to which they experience positive or negative emotions in academic life (Kim and Seo, 2015; Datu and King, 2018).

### Theoretical and practical implications

Theoretically, this study highlights the potential detrimental effects of hedonic orientation and urges a more comprehensive theory that can explain both positive and negative sides of the pursuit of happiness. Although the present study focused on students’ affective well-being and academic achievement, other aspects of well-being such as social acceptance and peer popularity are also crucial when defining the happiness of adolescents (Schwartz et al., 2010). Certain kinds of personality traits can be either adaptive or maladaptive depending on the criteria (Chen and Chang, 2012). Research has suggested that the pursuit of hedonia was negatively related to students’ satisfaction with school but positively related to students’ satisfaction with friends (Yang et al., 2017b). Moreover, a few recent studies have attempted to investigate potential moderators in the relationships between happiness orientation and actual level of well-being. For instance, Klipker et al. (2017) found that when confronted with daily hassles, hedonic orientation was negatively related to affective well-being in adolescents with low cognitive control; however, for adolescents with high cognitive control, strong hedonic orientation was related to improved affective well-being. In another study, Chen and Zeng (2021) demonstrated the moderating role of orientation priority. This refers to the relative importance placed on eudaimonic goals over hedonic ones, in the relationship between happiness orientation and individual well-being. Thus, hedonic orientation may produce either positive or negative outcomes, and the boundary conditions for when pursuing hedonia is beneficial or harmful require further exploration. In practice, guiding people in terms of whether or not to pursue a hedonic orientation is not a simple matter. Instead, practitioners need to be aware of the advantages and disadvantages of valuing happiness, and guide people to pursuit hedonia in a healthy and balanced way.

Notably, it should be cautious when generalizing the present findings to different populations. Recent research suggests that the associations of happiness orientation with individual well-being may not be the same in different cultures. The first reason is that, while the two constructs of hedonic and eudaimonic orientations have been validated across cultures, they can still carry different meanings for people from Western and Eastern countries, thus direct comparison of the constructs across cultures may be inappropriate (Odağ et al., 2016). Another reason is that hedonic motives are generally more valued by individualistic cultures than collectivistic ones (Joshanloo et al., 2021). Thus, hedonic orientation may be more maladaptive in collectivistic cultures such as China because people with strong hedonic motives will encounter more conflicts between personal values and cultural values (Joshanloo and Jarden, 2016). Moreover, research has suggested that Chinese culture emphasizes academic achievement, and students tend to internalize such goals (Zhu and Chang, 2019). Goal-congruent behaviors such as academic engagement will induce positive affect and incongruent behaviors such as procrastination will cause negative affect, which in turn may explain a stronger connection between hedonia and reduced affective well-being.

### Limitations and future research

This study has some limitations. First, while the study variables were assessed at different time points, which help to avoid common method bias, the data was ultimately correlational. Thus, the mediation analysis did not warrant a causal conclusion (Fiedler et al., 2011). Future studies should consider using longitudinal design or manipulating hedonic and eudaimonic motives to further determine the causal links between individuals’ happiness orientation, behavioral choice, and well-being outcomes. Second, this research was primarily based on self-reported measures, which are susceptible to biases such as social desirability and recall bias. Thus, other data resources, such as peer-reported and behavioral data are recommended in future research to provide convergent evidence.

## Conclusion

In summary, this study provides evidence of distinct associations of hedonic and eudaimonic orientations with both subjective experience and objective functioning. Pursuing eudaimonia is generally beneficial. However, pursuing hedonia may impair college students’ affective well-being and academic achievement by inhibiting academic behavioral engagement and increasing procrastination. The findings suggest that the pursuit of happiness may not always be successful and emphasize the disadvantages of the pursuit of hedonia.

## Data availability statement

The datasets presented in this study can be found in online repositories. The names of the repository/repositories and accession number(s) can be found below: https://osf.io/jnk23/?view_only=cf602ae806f246cfb6d349f940f0698b.

## Ethics statement

The studies involving human participants were reviewed and approved by Ethics Committee of Student Affairs Department, Zhejiang University of Finance and Economics. Written informed consent for participation was not required for this study in accordance with the national legislation and the institutional requirements.

## Author contributions

HC: methodology, formal analysis, and writing—original draft preparation. Both authors: conceptualization, writing—review and editing, read, and agreed to the published version of the manuscript.
